# A new strategy and system for the ex vivo ovary perfusion and cryopreservation: An innovation

**Published:** 2017-06

**Authors:** Mohamed Shehata Ali Mohamed

**Affiliations:** *M.D. Graduate, University of Cologne, Germany.*

**Keywords:** Ex vivo ovary perfusion, Ovary cryopreservation, Gonadotoxic cancer therapy, Fertility Preservation, Premature ovarian failure

## Abstract

Children and young adults, who suffer from cancer, receive gonadotoxic therapy, which destroys their fertile abilities after survival. Ovarian cryopreservation and transplantation provide the promising solution to this problem, where the ovary can be removed before the gonadotoxic therapy and reimplanted after patient's survival, where the ovary is to be cryopreserved during the period of the therapy. However, cryopreservation of the whole ovary is still facing great obstacles, namely the ischemic reperfusion injury and the defective cryopreservation related to the defective ability to universally deliver the cryopreservation/warming solutions through the ovarian vascular bed. Meanwhile, the currently applied technique of ovarian tissue cryopreservation provides limited follicular recovery because many follicles are lost until the development of revascularization post-transplantation. To solve the problems, an innovative system has been developed to insure immediate and universal delivery of the cryopreservation/warming solutions to the graft, in addition to keeping the graft under continuous perfusion before and after cryopreservation, minimizing any chance for microthrombi formation or ischemia-reperfusion. This innovative system can be applied in the following surgical and clinical interventions: 1) Allogeneic ovarian transplantation; 2) Preservation of fertility after systemic chemotherapy or bone marrow transplantation in young females, where the ovaries could be removed before the therapy and exposed to the adequate cryopreservation provided by the system till re-implantation after the patient's survival; 3) The system is also suitable for the corresponding applications on the testicles.

## Introduction

Ovarian cryopreservation aims at the preservation of fertility in young women subjected to gonadotoxic anti-cancer therapy. It is also important for women undergoing hematopoietic stem cells transplantation and other reasons of premature ovarian failure. Further indications could differ from country to another, based on the available medical regulations (1, 2). Ovarian cryopreservation can be performed using the slow freezing technique, where a programmable controlled temperature change can be applied, or the vitrification technique, where the ovarian tissue is equilibrated with suitable concentrations of cryoprotectants and directly plunged into liquid nitrogen (3, 4).

Recently, ovarian cryopreservation has been tried with animals (5) and humans (6), applying both slow freezing (7-14) and vitrification techniques (14-22), with the results of vitrification are comparable to those of slow freezing (14). Ovarian cryopreservation is considered important rather than mature oocyte or embryo cryopreservation because it preserves the hormonal and fertility functions.

The ovary is composed of the cortex, which is the outer part and contains tightly packed connective tissue, and the medulla, which is central and highly vascular. The majority of the follicles, in varying stages of maturation, are located in the cortex, including the primordial follicles that require the signal for further development. In the currently applied protocols, the cortex is dissected from the medulla and subjected to cryopreservation, where many primordial follicles are present. This technique is associated with less injury rather than the cryopreservation of individual oocytes and or embryos. This may be related to the smaller size and the slower metabolic rates of the primordial follicles,the absence of zona pellucida and the in-vivo follicular maturation following transplantation (25). Accordingly, the ovarian cortex is the actual part subjected to cryopreservation, following the sharp dissection from the medulla.

After the successful cancer therapy, the thawed ovarian cortex is transplanted close to the fallopian tube, allowing the in-vivo maturation of the primordial follicles and the reestablishment of the hormonal and the fertilizing functions of the ovary (26-30).

**Current techniques in ovarian cryopreservation**

Cryopreservation is the process, where biological materials, cells and tissues are preserved in sub-zero temperatures to be retrieved through warming in the subsequent future. At such very low temperatures, the enzymatic activities that could damage the cell are stopped. The success of the cryopreservation depends on the use of certain compounds that protect the cells (hence, called cryoprotectants) as well as the cooling rate (31).

Cryoprotectants can be divided into two major categories; Permeable cryoprotectants that have the ability to following cancer therapy. In addition, the cryopreservation of oocytes or embryos is not always possible before cancer therapy in cases of prepubertal girls, hormone- sensitive tumors, or because of the lack of a partner and or enough time (23, 24).

Cryoprotectants can be divided into two major categories; Permeable cryoprotectants that have the ability to penetrate the cell membrane and, accordingly, protect against the intracellular ice formation. The use of permeable cryoprotectants is associated with significant toxicity and extra-stress on the cell during their addition and removal, before freezing and after thawing, respectively (32). Non-permeable cryoprotectants that cannot penetrate the cell membrane and, accordingly, protect against the extracellular ice formation (33).

Ovarian cryopreservation could be achieved through the slow freezing (programmable) rate or the ultra-rapid freezing rate (vitrification). Vitrification is the technique, where the cells/tissues are frozen at an ultra-rapid rate by direct plunging into liquid nitrogen, where the cells suspension change into a glass-like solidification, avoiding ice crystal formation (34). Rapid rates of freezing do not allow the permeable cryoprotectants to exert their protective actions, which essentially require slow cooling rate. Meanwhile, the non-permeable cryoprotectants stabilize the cells during the ultra-rapid freezing (35).

Slow freezing of the ovarian tissue starts with the cutting of the ovarian cortex into strips, as an initial step. An example of a protocol could be as following, where the cortical strips are suspended in a cryoprotective solution at 4^o^C. The strips are then to be transferred to 2-mL cryovials, containing 0.8 mL cryoprotective solution that are to be cooled in a programmable freezer from 0C to -8^o^C at -2^o^C /min, then to be cooled to -40^o^C at -0.3^o^C /min. This is followed by cooling to -140^o^C at -30^o^C /min and transfer to liquid nitrogen (-196^o^C) for storage. 

The programmable freezing might be performed manually by placing the cryovials in the nitrogen vapor before plunging into the liquid nitrogen, where the temperature of the vapor depends on the distance between the vials and the surface of the liquid as well as on the amount of the used liquid nitrogen. However, the control of the cooling rates in this case would be difficult (36). For thawing, the cryovials are simply placed in room temperature for 2 min, and then in a 37^o^C water bath till the ice completely melt. Before transplantation, the ovarian tissue is placed in plastic petri dishes containing minimal essential medium (MEM), supplemented with Glutamax, for washing. The washing is to be performed, at least, for three times (5 min each) to get rid of the cryoprotectants (36). Vitrification of the ovarian tissue starts with the cutting of the ovarian cortex into cubes, as an initial step. Two protocols could be examples for ovarian tissue vitrification; 

Protocol 1, where the vitrification solution, which contains 20% dimethylsulfoxid (DMSO), 20% ethylene glycol in MEM, supplemented with 25 mg/mL human serum albumin (HSA), is used. The ovarian cubes are to be equilibrated in 25% vitrification solution for 5 min (at 4^o^C), followed by equilibration in 50% vitrification solution for another 5 min (at 4^o^C), and finally in 100% vitrification solution for 10 min (at 4^o^C). Following equilibration, the ovarian cubes are to be placed on an aseptic absorbent gauze to remove the remaining vitrification solution. The cubes are then transferred to a stainless steel box, partially immersed in liquid nitrogen. Now the ovarian fragments are vitrified and are to be placed in pre-cooled cryovials for long term storage in liquid nitrogen (37).

Protocol 2, where the vitrification solution is composed of 10% dimethylsulfoxid (DMSO), and 26% ethylene glycol in MEM, supplemented with 20 mg/mL HSA, 2.5% polyvinylpyrrolidone and 1 mol/L sucrose. The ovarian cubes are to be equilibrated in 25% vitrification solution (for 5 min), then in 50% vitrification solution (for 5 min), and finally in 100% vitrification solution (for 1 min), at room temperature. After equilibration, the cubes are to be placed on an aseptic absorbent gauze at room temperature, in order to remove the remaining vitrification solution. The cubes are then to be placed in open cryostraws, and to be plunged into liquid nitrogen. Now the ovarian fragments are vitrified and are to be placed in pre-cooled cryovials for long term storage in liquid nitrogen (33, 38).

For warming of the vitrified ovarian tissue, the ovarian strips or cubes are taken out from the cryovials and placed into warming solution 1 at 37^o^C, which is composed of 1 mol/L sucrose in MEM, supplemented with 25 mg/mL HSA. The tissue is to be incubated in this solution for about 15 sec, and then to be transferred to the warming solution 2 (which has the same composition of warming solution 1, apart from 0.5 mol/L sucrose concentration), followed by warming solution 3 (which has the same composition of warming solution 1, apart from 0.25 mol/L sucrose concentration), and warming solution 4 (which has the same composition of warming solution 1, apart from 0 mol/L sucrose concentration), for 5 min at 37^o^C each (33, 37, 38).

Several studies have reported successful results of transplantation of cryopreserved ovarian tissues of both techniques, with the results of vitrification being superior or, at least, comparable to those of slow freezing (33). However, and regardless of the technique of cryopreservation, there are two major problems hindering the transplantation of the ovarian tissue and limiting the graft survival; the cryoinjury in the case of cryopreservation, and the ischemic reperfusion injury. 

As the ovarian cortex is composed of tightly packed connective tissue, where it depends on the underlying highly vascular ovarian medulla for getting the necessary oxygen and nutrient supplies, it takes a relative longer time (2-3 days) to re-establish adequate vascular connections with the underlying tissue following transplantation, which leads practically to some degree of follicular atrophy and failure of long term graft survival. This is why the peritoneal grafting of the ovarian tissue has been associated with better results than the subcutaneous grafting, as the revascularization in the peritoneal case is better. 

However, in all cases, a large number of follicles would be ultimately lost until the neovascularization occurs, accordingly, there would be a high risk of graft failure (39, 40). Many strategies have been tried to minimize the post-transplantation ischemic ovarian damage, however, these strategies lack the standardization, the reproducibility and the long term success (41). Examples of those strategies are:

- Mechanical induction of neoangiogenesis, through the induction of tissue injury and grafting the ovarian tissue on the being formed granulation tissue (42).
- The use of antioxidants (43-45).
- The use of neoangiogenesis - inducing growth factors (46).
- The use of hormonal therapy (47).

Nevertheless, a promising solution for that problem is the whole ovary transplantation, where the ovarian vascular pedicle is anastomosed during transplantation, using microvascular surgery (48).

Although this strategy provides a very promising theoretical solution, which can be applied for both fresh and frozen/thawed ovaries, and can significantly minimize the ischemic injury after transplantation, there are still major troubles facing it (49-52):

1. The ability to efficiently cryopreserve the whole ovary, instead of the ovarian tissue fragments. The main obstacles of this procedure are the intravascular thrombosis and the inability to efficiently deliver the cryopreservation and warming solutions simultaneously to the whole ovary.
2. The complexity of the surgical procedure and the ischemic reperfusion injury associated with all solid organs transplantation.

To overcome those problems, the following innovative technique is introduced to improve the ovarian cryopreservation and transplantation clinical outcome.

**The innovative ex vivo ovary perfusion model of Shehata**

In the present technique, the donor ovary, which belongs to the cancer patient herself or the matching donor for cases of ovarian failure, is removed by a laparoscopic or an open surgical approach, where the vascular pedicle of the ovary is accessed. The ovarian artery is approached as proximal as possible, and catheterized after giving off its branches. Similarly, the ovarian vein is approached as distal as possible, and catheterized before receiving its tributaries.

**Figure 1:**
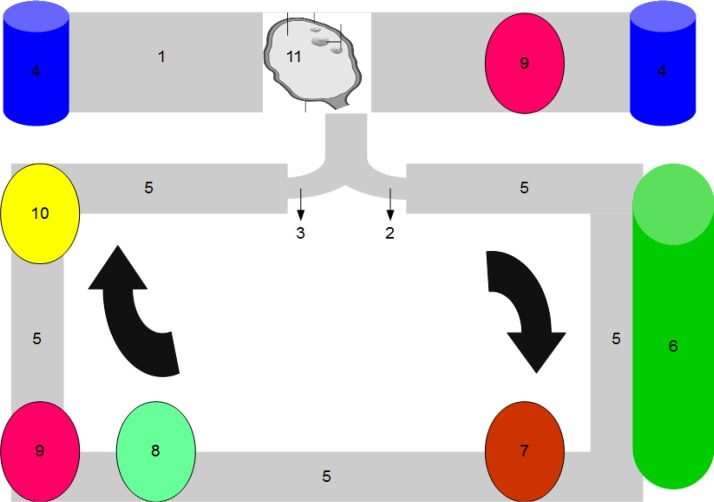
Diagrammatic representation of the ex vivo ovary perfusion system:

Now, the donor ovary can be surgically removed, while both the vascular input and output are catheterized, however, before removal, in-vivo perfusion through the catheters is to be performed for few minutes, to avoid ischemia and any risk of the microthrombi formation. The arterial and venous catheters are connected to a circuit of perfusion, composed of the following (Figure 1):

- A box to enclose the ovary and can be used as a sealed cryovial.
- *The box enclosing the ovary can work as a sealed cryovial, which allows the passing of the perfusion input and output catheters during perfusion, and the complete sealing during liquid nitrogen storage*
- The circuit itself is made of perfusion tubes, connected to the arterial and venous catheters.
- Reservoirs *to introduce and remove the perfusates (perfusion supplemented medium, cryopreservation and warming solutions)*

A pulsatile or continues flow centrifugal pump, providing flow equivalent to the estimated physiological ovarian artery flow of the donor (pre-cryopreservation) and of the recipient (post-cryopreservation).

- A set of leukocytes and cytokines filters.
- A temperature adjustor that can control the temperature of the perfusate.
- *A gas exchanger to remove CO*_2_ *and provide O*_2_ *,* to maintain these gases in the perfusate at the physiological levels.

The whole system will be placed on a portable mobile base, to allow the transport of the ovary from the donor hospital to the cryopreservation center and or the recipient hospital while perfused, in order to avoid warm ischemia. 

The introduced procedure starts with the surgical retrieval of the donor ovary, where vascular catheterization and immediate perfusion begins. The used perfusate can vary according to the protocol used, for instance, minimal essential medium (MEM) supplemented with HSA or human tubal fluid medium supplemented with serum substitute supplement (SSS). Further supplementation could be considered according to the used protocol, such as ascorbic acid, antioxidants, hormones, growth factors, antibiotics, heparin, etc. 

The immediate and continuous perfusion minimizes the risk of the microthrombi formation, however, thrombolytic medications could be supplemented to the perfusate, in order to dissolve any formed microthrombi. At this stage, the donor ovary has not manifested significant ischemia or oxygen or nutrient deprivation, and the vascular bed is clearly accessible. In case of fresh ovary transplantation, the ovary could be kept perfused under normothermic or hypothermic conditions till transplantation and intraoperative vascular anastomosis, allowing the chance of gross and or microscopic assessment of the graft, when needed. In this case, the ischemic reperfusion injury would be significantly minimized.

However, in case the cryopreservation of the ovary is planned, the cryopreservation solution (either for slow freezing or for vitrification) can be introduced into the circuit to simultaneously fill the cleaned vascular bed of the ovary and the plastic box around the graft. This ability, together with the presence of temperature adjustors, allow the application of the cryopreservation or vitrification protocol of interest (as previously described), where at the end, the graft can be stored in liquid nitrogen. 

These abilities of the system allow, as well, the application of the warming protocols (as previously described), where the graft can be further perfused till the surgical transplantation, minimizing the ischemic reperfusion injury.

1. A box to enclose the ovary and works as a sealed cryovial
2. Ovarian vein stump connected to the circuit, using a special cannula or catheter (perfusion output)
3. Ovarian artery stump connected to the circuit, using a special cannula or catheter (perfusion input)
4. Reservoirs for filling the ovary-containing box with medium, the cryoprotective, vitrification, and warming solutions during cryopreservation
5. The perfusion circuit
6. Perfusate reservoir
7. Centrifugal pump (pulsatile or continuous flow)
8. Set of leukocytic and cytokines filters
9. Temperature adjustor
10. *A gas exchanger to remove CO*_2_ *and provide O*_2_ *,* to maintain these gases in the perfusate at the physiological levels
11. The ovary graft inside the cryovial/ perfusion box

**The innovation in Shehata's ovary system**

As previously discussed, the whole ovary cryopreservation has been introduced and experimentally tested.41 In addition, the idea of ex vivo perfused ovary was used before in some experimental studies for the ex vivo culture of the whole ovary, in order to allow the ex vivo oocyte maturation and development.53 However, to the best of my knowledge, it is the first time to introduce the ex vivo perfusion technique as a procedure that should precede and follow the cryopreservation of the ovary, in order to overcome the obstacles that limit the success of the technique. 

Moreover, the introduced system is very unique, allowing the application of both ex vivo perfusion and cryopreservation in one system, which ensures better and simultaneous delivery of the cryoprotective and warming solutions to the inside and the outside of the organ, allowing for better outcome of the procedure. Some advantages of the system are listed in table I.

**Table I:** some advantages of the Shehata's system for ex vivo ovary perfusion and cryopreservation

The system allows the ex vivo perfusion of the ovary: In case of allogenic ovary transplantation, minimizing the ischemic reperfusion injury In case of cryopreservation, allowing the easy and universal introduction and removal of the cryopreservation and warming solutions
The system allows the ex vivo perfusion of the ovary after thawing and before transplantation, which allows the conditioning of the organ before reimplantation
The preservation and transplantation of the whole ovary is expected to: Be better than ovarian tissue tissue transplantation, Preserve the fertility and the endocrine functions of the ovary, Protect against the loss of ovarian follicles, while waiting for adequate neovascularisation
The system is mobile, allowing the transfer of the organ while under perfusion and reconditioning
The system is also suitable for corresponding applications on the testicles

**Clinical applications and intended use of the proposed system**

The proposed system can be applied in the following surgical and clinical interventions:

1. Allogeneic ovarian transplantation, where the system will allow the ex vivo organ perfusion, minimizing the ischemic reperfusion injury and allowing graft investigation and conditioning before transplantation. In this case, the set of leukocytic and cytokine filters are included to filter the donor leukocytes and minimize the hazards of the ischemic reperfusion injury.54

2. Localized treatment of ovarian cancers without exposing the patient to the systemic hazards of chemotherapy and radiotherapy, where the affected ovary could be surgically isolated from the patient's body and circulation, and kept under perfusion in the system, while localized and highly concentrated doses of chemo- and or radiotherapy could be applied to target the cancer cells within the ovary (to preserve fertility, if some healthy tissue and follicles could survive the therapeutic techniques).

3. Preservation of fertility after systemic chemotherapy or bone marrow transplantation in young females, where the ovaries could be removed before the therapy and exposed to the adequate cryopreservation provided by the system till reimplantation after the patient's survival. 

4. The system is also suitable for corresponding applications on the testicles.

## Conflict of interest

The intellectual properties and the system included in this manuscript belong solely to the author. All rights are preserved solely for the author. Reproduction or use of any of the included intellectual properties requires the written permission of the author. No funding was provided for the development of this work. The author welcomes funding cooperation for experimental and clinical studies.
